# Supplementary material for: The genome sequence of touch-me-not balsam, Impatiens noli-tangere L. (Ericales: Balsaminaceae)
Source: Wellcome Open Res. 2026 Sep 21;11:718. [Version 1] doi: 10.12688/wellcomeopenres.27873.1 (PMC13675621; doi:10.12688/wellcomeopenres.27873.1)
Supplement: Peer review history

# Reviewer report: Reviewer response for version 1

**DOI:** 10.21956/wellcomeopenres.30670.r171203

**Published:** 2026-10-16

**Authors:** Harpreet Kaur

**Affiliations:** 1 University of Minnesota, St. Paul, Minnesota, USA

The manuscript titled “The genome sequence of touch-me-not balsam, *Impatiens noli-tangere* L. (Ericales: Balsaminaceae)” presents a phased genome assembly for this species, with one haplotype scaffolded to chromosome level. Overall, the manuscript is well presented, and the assembly provides a valuable genomic resource. I have a couple of minor suggestions and queries:

1. **Background:** Please include information on the species’ pollination and reproductive biology, particularly whether it is predominantly self-pollinating, cross-pollinating, if known. This would provide useful context for the reported heterozygosity estimate and potential challenges associated with genome assembly and haplotype phasing.
2. **Genome sequence report:** Please clarify why only haplotype 1 was scaffolded and curated to chromosome level, whereas haplotype 2 remains at scaffold level. Given that Hi-C data were available and used for haplotype phasing, was this a deliberate choice, or were there technical limitations that prevented chromosome-level scaffolding of haplotype 2?

Thank you!

Are the datasets clearly presented in a usable and accessible format, and the assembly and annotation available in an appropriate subject-specific repository?

Yes

Are sufficient details of the sequencing and extraction, software used, and materials provided to allow replication by others?

Yes

Are the rationale for sequencing the genome and the species significance clearly described?

Yes

Are the protocols appropriate and is the work technically sound?

Yes

Reviewer Expertise:

Alfalfa Genomics and Breeding

I confirm that I have read this submission and believe that I have an appropriate level of expertise to confirm that it is of an acceptable scientific standard.

# Reviewer report: Reviewer response for version 1

**Published:** 2026-09-21

**Authors:** Automated Benchmarking

**Affiliations:** 1 Wellcome Sanger Institute, Hinxton, UK

As this article is a Genome Note, Automated Benchmarking has been used to determine whether the reported assembly passes four defined checks established by the Earth BioGenome Project: the raw and assembled data are publicly available, the Earth BioGenome Project three-part metric is met, the k-mer completeness exceeds 90%, and the BUSCO score passes the stated threshold. For Impatiens noli-tangere (ddImpNoli1), all four checks passed. Data available: pass. ENA study PRJEB77495, raw-read accessions ERR13362649, ERR13362650, ERR13363428, and ERR13962523, and assembly accessions GCA_965213615.1 and GCA_965213605.1 are public. Earth BioGenome Project three-part metric: pass. Haplotype 1 is reported as 6.C.Q66, meeting the 6.C.Q40 reference standard. k-mer completeness: pass. The combined haplotype assemblies have 98.72% k-mer completeness, exceeding the Earth BioGenome Project target of 90%. BUSCO: pass. Haplotype 1, which is proposed as the reference assembly for this species, has 94.5% complete BUSCOs (87.0% single-copy and 7.5% duplicated). BUSCO software version: 5.7.1; lineage dataset: eudicots_odb10. It meets the plant-specific target of greater than 90% complete BUSCOs. This decision uses values reported in the Genome Note and public accession records.

Reviewer Expertise:

NA

I confirm that I have read this submission and believe that I have an appropriate level of expertise to confirm that it is of an acceptable scientific standard.
